# *Plasmodium falciparum* Mitochondrial Complex III, the Target of Atovaquone, Is Essential for Progression to the Transmissible Sexual Stages

**DOI:** 10.3390/ijms25179239

**Published:** 2024-08-26

**Authors:** Pradeep Kumar Sheokand, Sabyasachi Pradhan, Andrew E. Maclean, Alexander Mühleip, Lilach Sheiner

**Affiliations:** Wellcome Centre for Integrative Parasitology, School of Infection and Immunity, University of Glasgow, Glasgow G12 8TA, UK; pradeepsheokand6@gmail.com (P.K.S.); sabyasachi.pradhan@glasgow.ac.uk (S.P.); andrew.maclean@glasgow.ac.uk (A.E.M.); alexander.muhleip@glasgow.ac.uk (A.M.)

**Keywords:** *Plasmodium*, mitochondrion, mETC, complex III, Rieske, gametocytes

## Abstract

The *Plasmodium falciparum* mitochondrial electron transport chain (mETC) is responsible for essential metabolic pathways such as de novo pyrimidine synthesis and ATP synthesis. The mETC complex III (cytochrome *bc*_1_ complex) is responsible for transferring electrons from ubiquinol to cytochrome *c* and generating a proton gradient across the inner mitochondrial membrane, which is necessary for the function of ATP synthase. Recent studies have revealed that the composition of *Plasmodium falciparum* complex III (PfCIII) is divergent from humans, highlighting its suitability as a target for specific inhibition. Indeed, PfCIII is the target of the clinically used anti-malarial atovaquone and of several inhibitors undergoing pre-clinical trials, yet its role in parasite biology has not been thoroughly studied. We provide evidence that the universally conserved subunit, PfRieske, and the new parasite subunit, PfC3AP2, are part of PfCIII, with the latter providing support for the prediction of its divergent composition. Using inducible depletion, we show that PfRieske, and therefore, PfCIII as a whole, is essential for asexual blood stage parasite survival, in line with previous observations. We further found that depletion of PfRieske results in gametocyte maturation defects. These phenotypes are linked to defects in mitochondrial functions upon PfRieske depletion, including increased sensitivity to mETC inhibitors in asexual stages and decreased cristae abundance alongside abnormal mitochondrial morphology in gametocytes. This is the first study that explores the direct role of the PfCIII in gametogenesis via genetic disruption, paving the way for a better understanding of the role of mETC in the complex life cycle of these important parasites and providing further support for the focus of antimalarial drug development on this pathway.

## 1. Introduction

Malaria is a deadly disease that causes significant morbidity and mortality around the globe, with 249 million cases and ~608,000 deaths reported in 2022 alone [1]. It is caused by mosquito-transmitted parasites of the genus *Plasmodium* spp., with *Plasmodium falciparum* being responsible for most fatal cases in humans. The parasite has a complex life cycle with several life stages parasitizing different tissues in the host and in the vector [2]. In the blood of the host, the parasite life stages can be divided into the asexual stages, responsible for the disease symptoms, and the sexual, gametocyte stage. Mosquitos can take up the gametocytes during a blood meal, thus initiating transmission. Drugs targeting both these stages exist and are used as one of the strategies for malaria control [3,4]. The rapid emergence of drug resistance, however, threatens the current first-line therapies and highlights a need to develop new compounds [5]. A better understanding of pathways that are known to be drug targets may contribute to this goal.

One of these pathways is the *Plasmodium* mitochondrial electron transport chain (mETC). This pathway harnesses electrons from different metabolic pathways via a series of dehydrogenases, including malate quinone oxidoreductase (MQO), dihydroorotate dehydrogenase (DHODH), succinate dehydrogenase (complex II), and a type II NADH dehydrogenase (NDH2). The electrons are transferred between complexes of the mETC via the mobile carriers, ubiquinone, which transfers electrons to complex III (cytochrome *bc*_1_ complex), and cytochrome *c*, which transfers them to complex IV (cytochrome *c* oxidase). At complex IV, these electrons reduce oxygen to water. The main function of mETC-mediated electron transfer in asexual parasites is the electron harnessing necessary for the pyrimidine synthesis pathway [6]. Additionally, the movement of the electrons through the mETC complexes III and IV is coupled to the pumping of protons, which generates a proton gradient across the inner mitochondrial membrane. This proton gradient is utilized by ATP synthase (complex V) to generate ATP. This process is collectively named oxidative phosphorylation (OXPHOS). While not essential for asexual blood stages (which generate most of its ATP via cytoplasmic glycolysis), ATP synthase becomes essential for the transmission stages [7]. Therefore, the role of mETC is essential in both critical stages of the parasite life cycle.

The *Plasmodium falciparum* mETC complex III (PfCIII) is a well-established drug target for the clinically used drug atovaquone (ATQ) [8,9,10] and for inhibitors undergoing pre-clinical trials such as the series of endochin-like quinolones (ELQs) [11,12,13]. In mammals, three subunits compose the catalytic core of complex III: (1) the mitochondrially encoded cytochrome b (cytb), which spans the inner mitochondrial membrane and thus connects the two substrate binding sites named Qo and Qi; (2) cytochrome c1 (cytC1) and (3) Rieske. The last two together mediate the transfer of electrons between the two mobile carriers. The human complex III consists of eight further non-catalytic subunits [14]. PfCIII is proposed to consist of 12 subunits, which include homologs of cytb, cytC1, and Rieske, but not all eight non-catalytic subunits found in humans have homologs [15]. Moreover, three additional apicomplexan-specific subunits (PfC3AP1, PfC3AP2, PfC3AP3) are proposed to be part of PfCIII [15]. Despite its divergence and importance as a drug target, the composition and function of PfCIII are not fully understood. In this study, we provide support for the previously proposed divergent PfCIII composition through co-immunoprecipitation, localization, and gel migration of endogenously tagged proteins while focusing on one conserved (PfRieske) and one apicomplexan-specific (PfC3AP2) subunits. Using conditional knockdown, we demonstrate that PfRieske is required for PfCIII function and confirm that the complex is required for asexual blood stage survival whereby PfRieske-depleted parasites show a defect in pyrimidine synthesis and become hypersensitive to mETC inhibitors, in line with previous reports of PfCIII disruption [10,16]. We further found that PfRieske depletion resulted in a defect in gametocyte stage progression, which coincides with a defect in mitochondrial cristae formation. This work provides support to the predicted role of PfCIII in the mitochondria of asexual and sexual *Plasmodium* stages and highlights the pathways it contributes to.

## 2. Results

### 2.1. PfRieske and PfC3AP2 Are Part of Plasmodium falciparum Complex III (PfCIII)

**Figure 1:**
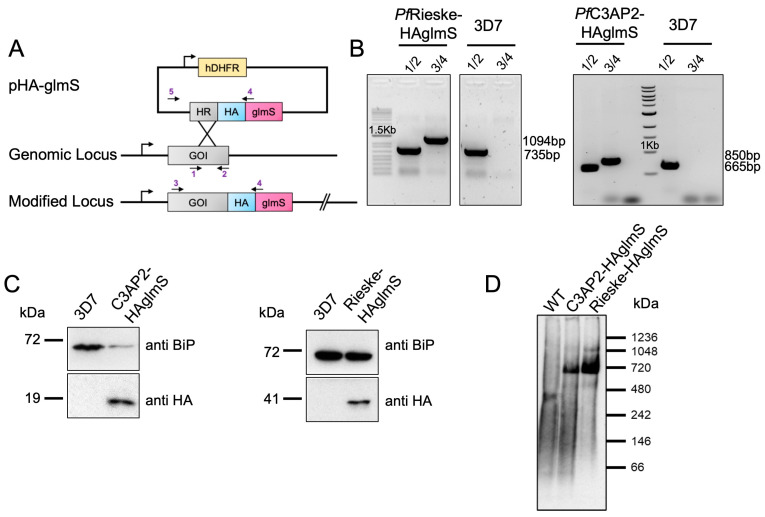
Tagging analysis and immunoprecipitation of the PfRieske and PfC3AP2: (**A**) Schematics of the endogenously C-terminal tagging strategy. (**B**) PCR analysis confirming the regulatable cassette integration using primers corresponding to the scheme in (**A**). (**C**) SDS-PAGE western blot of endogenously tagged PfRieske and PfC3AP2 using anti-HA antibody and anti-BiP as a loading control. (**D**) BN-PAGE Western blot of both tagged proteins using the anti-HA antibody.

Recent studies of the apicomplexan mETC complex composition via complexome profiling in both *Plasmodium falciparum* and *Toxoplasma gondii* have shown that their complex III consists of 12 and 11 subunits, respectively [15,17]. Both studies further identified new proteins that have no clear homologs in the mammalian complex [15,17]. In *Plasmodium,* three new proteins (PfC3AP1, PfC3AP2, PfC3AP3) are annotated as putative PfCIII subunits. To provide support for this divergent subunit composition, we created tagged lines of two subunits, the parasite-specific PfC3AP2 and the conserved PfRieske. While engineering the tagged lines, we included the glucosamine-6-phosphate-activated ribozyme (glmS) system for inducible gene depletion [18]. Thus, we generated Hemagglutinin (HA) tagged PfC3AP2-HA-glms and PfRieske-HA-glmS transgenic parasite lines whereby the C-terminal of each gene is fused with the HA-glmS tag, and the fusion proteins are expressed under the native promoter of the respective genes (the construct and resulting genetic manipulation are shown in the scheme in Figure 1A). The correct integrations of the HA-glmS tags in the target loci were confirmed by PCR (Figure 1B) and Western blot analysis (Figure 1C). Native PAGE and Western blot analysis, used previously to resolve mETC complexes in the related apicomplexan *T. gondii* [17], further showed that both the proteins migrated at the same size of ~730 kDa (Figure 1D) in line with the predicted size of a PfCIII dimer based on its proposed composition [15]. This provided validation that both proteins are part of the complex. To provide further evidence of interaction between these proteins and other PfCIII subunits, we performed immunoprecipitation experiments with the PfRieske-HA-glmS transgenic parasite line (Figure S1) followed by mass spectrometry analysis (Table S1). We found 7 of the expected 12 subunits in one experiment and 10 in another (Table 1) including PfC3AP2. No subunits of PfCIII were found in a further experiment performed on the parental line, demonstrating the specificity of the experiment. These findings provide support for the proposed composition and further validate that both subunits are part of PfCIII.

**Figure 2:**
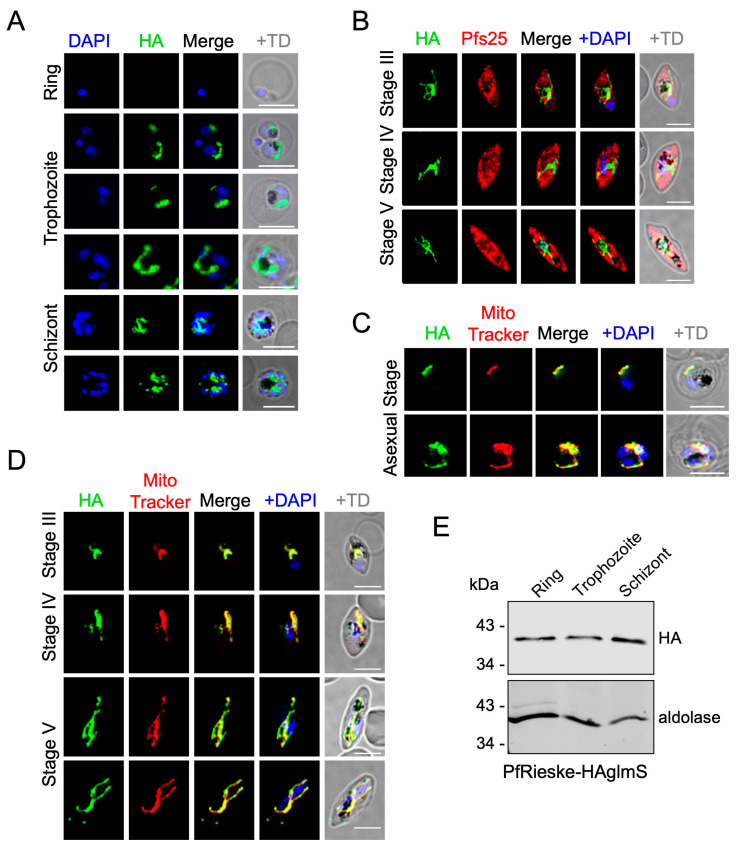
Expression and subcellular localization of PfRieske in *P. falciparum*. (**A**,**B**) Immunofluorescence assay of PfRieske, visualized with anti-HA, in asexual blood stages (**A**) and in gametocytes (**B**). (**C**,**D**) Immunofluorescence assay of PfRieske visualized with anti-HA and co-stained with MitoTracker-Deep-Red showing colocalization in both asexual (**C**) and sexual (**D**) blood stage parasites. Scale bar: 5 µm. (**E**) SDS-PAGE and western blot analysis to detect PfRieske-HA across the different developmental stages of the asexual blood stage cycle. Aldolase was used as a loading control.

**Figure 3:**
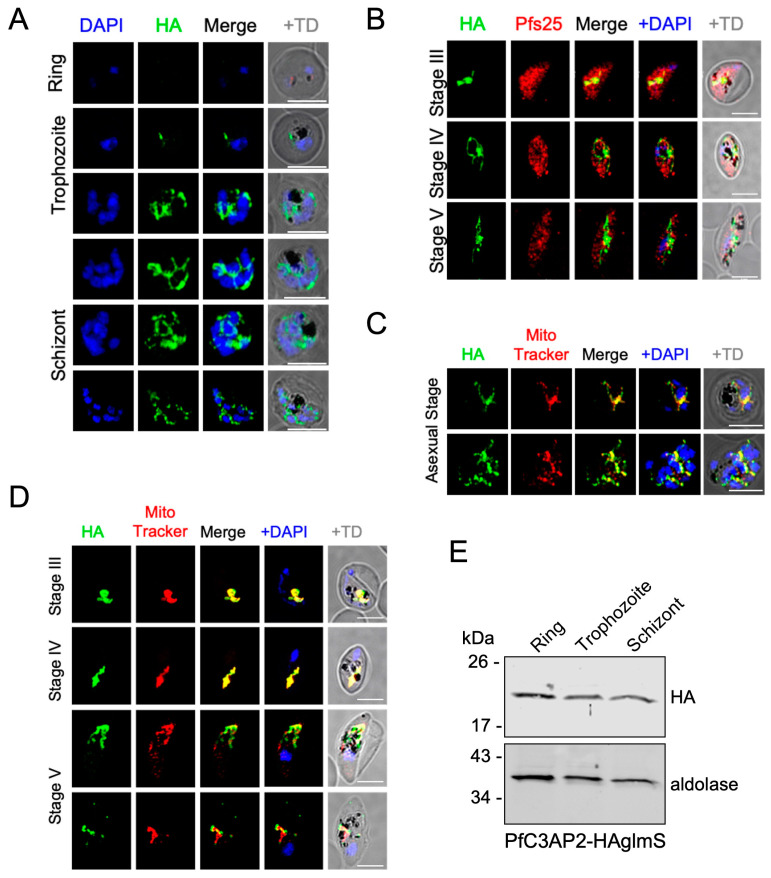
Expression and subcellular localization of PfC3AP2 in *P. falciparum*. (**A**,**B**) Immunofluorescence assay of PfC3AP2, visualized with anti-HA, in asexual blood stage (**A**) and in gametocytes (**B**). (**C**,**D**) Immunofluorescence assay of PfC3AP2 visualized with anti-HA and co-stained with Mito Tracker-Deep-Red showing co-localization in both asexual (**C**) and sexual (**D**) blood stage parasites. Scale bar: 5 µm. (**E**) Western blot showing the expression of the PfC3AP2 across the different developmental stages of the asexual blood stage cycle. The membranes were probed with an anti-HA antibody, and anti-aldolase was used as a loading control.

Next, we used the endogenous tags to analyze the localization of both proteins using an anti-HA antibody via immunofluorescence of culture at ~5–10% parasitemia fixed in 4% paraformaldehyde and 0.05% glutaraldehyde. We observed the signal in both asexual blood stages as well as in gametocyte stages (marked with the gametocyte marker Pfs25). The signal could not be detected in asexual ring stages, potentially due to the small size of their mitochondria impairing detection (we have repeated these IFAs three times with the same observation). However, the signal manifested as a tubular shape in trophozoites and marked a branched morphology in schizonts (Figure 2A and Figure 3A). In gametocytes, the signal manifests as an elongated and tubular structure (Figure 2B and Figure 3B). These observations are in line with mitochondrial morphology [19,20]. In agreement with this, both the proteins co-localized with MitoTracker-Deep-Red, which confirms the predicted localization of these proteins to the mitochondria (Figure 2C,D and Figure 3C,D). Western blot analysis confirmed the expression of both proteins across the different stages of the asexual blood cycle, including the ring stages, whereby we suspect that the higher sensitivity of the western blot assay enabled detection (Figure 2E and Figure 3E).

### 2.2. PfRieske Is Essential for Parasite Growth

**Figure 4:**
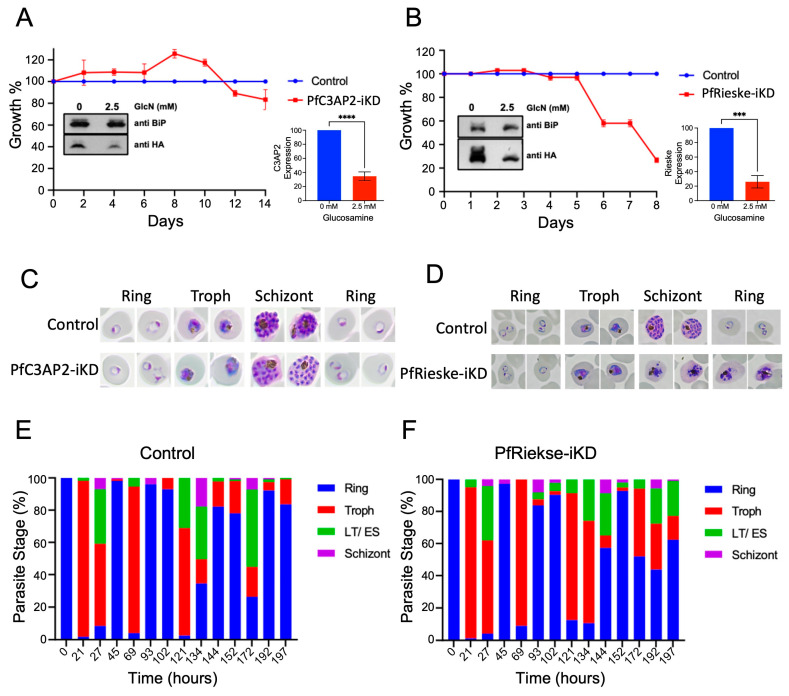
Inducible knockdown shows that PfRieske is essential for the asexual blood stage parasite growth: (**A**,**B**) Growth curves of PfC3AP2-iKD *n* = 2 (**A**) and PfRieske-iKD *n* = 3 (**B**) transgenic parasites growing in presence and absence of the 2.5 mM glucosamine (GlcN). Insets show western blots of the parasite cell lysate grown in the presence or absence of GlcN probed with anti-HA and anti-BiP antibodies of the parasite cell lysate grown in GlcN presence and absence condition. Error bars are SEM. The unpaired two-tailed *t*-test was used to calculate the statistics for the mean. *** *p* < 0.001, **** *p* < 0.0001. (**C**,**D**) Representative images of Giemsa-stained smears of the third cycle of the C3AP2-iKD (**C**) and PfRieske-iKD (**D**) parasites grown in the presence or absence of GlcN. Magnification 100×. (**E**,**F**) Bar graph showing quantification of the life stage progression of the PfRieske-iKD parasites in the absence (**E**) and presence (**F**) of GlcN *n* = 2 for both. LT/ES—late trophozoite/early schizonts.

The importance of PfCIII for *Plasmodium* survival is well established both based on numerous inhibitor sensitivity and resistance mutant analyses [21,22,23,24,25] and, more recently, through the study of the subunit cytC1 [16]. However, the impact of PfCIII disruption on parasite biology has not been fully characterized. To address this gap, we focused on the two subunits we validated here and attempted their genetic disruption. We used the HA-glmS lines to induce a knockdown of each gene. Western blot analyses using the HA tag showed a significant decrease in the levels of both PfC3AP2 and PfRieske when parasites were grown in the presence of glucosamine (GlcN) (Figure 4A,B). To address the effect of these knockdowns on parasite growth, we cultured the PfRieske-HA-glmS and PfC3AP2-HA-glmS parasites in presence of the GlcN (from here on, these conditions are named PfRieske-iKD and PfC3AP2-iKD, respectively) and monitored the growth over four and eight 48 h-long intra-erythrocytic cycles (IDCs), respectively. We did not observe a growth defect in PfC3AP2-iKD even after seven IDCs (Figure 4A). On the other hand, a severe growth defect at three IDCs was seen upon PfRieske depletion (Figure 4B). Giemsa-stained PfRieske-iKD parasites seem morphologically similar to their parental control for two IDCs, and the growth defect was observed at the late-trophozoite-to-early-schizont stage in the third IDC, which is confirmed by stage quantification analysis of the PfRieske-iKD parasites compared with the parental control (Figure 4C–F). These data indicate that the PfRieske subunit is essential for asexual parasite development. 

### 2.3. Loss of PfRieske Results in Hypersensitivity to mETC Inhibitors and to Membrane Depolarization by Proguanil

**Figure 5:**
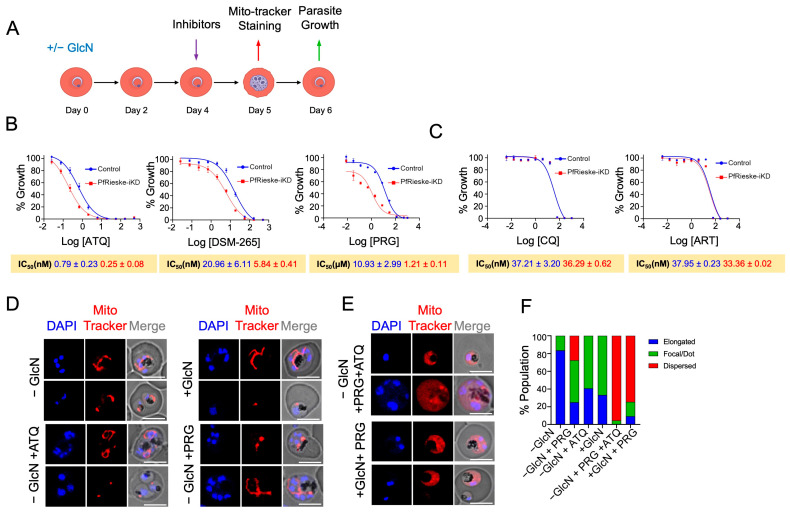
Depletion of PfRieske results in mETC failure: (**A**) Schematic representation of our experimental setup for the membrane potential and hypersensitivity assays. (**B**) IC50 analysis reveals hypersensitivity of GlcN-treated parasites (PfRieske-iKD) to the mETC inhibitors atovaquone (ATQ), DSM-265, and proguanil (PRG). (**C**) IC50 analysis confirms no change in the sensitivity of GlcN-treated parasites (PfRieske-iKD) to drugs used as a negative control for (**B**) Chloroquine (CQ) and Artemisinin (ART). The graphs in B and C are each representative of *n* = 2 independent experiments, and the numbers are the mean of the technical triplicates performed in each of the two. (**D**,**E**) confocal fluorescent microscopy images of MitoTracker-Deep-Red stained parasites grown in the presence or absence of glucosamine, atovaquone, and proguanil (±GlcN/ATQ/PRG). Combinations that do not affect the membrane potential are shown in (**D**) as controls, while treatment with atovaquone and proguanil or upon PfRieske depletion and proguanil treatment are shown in (**E**). Scale bar: 5 µm (**F**) Quantification of the mitochondrial signal in each of the conditions in (**D**,**E**). The data shown are representative of the *n* = 2 independent experiments.

Several compounds are well-established inhibitors of enzymes involved in the transport of electrons in the *Plasmodium* mitochondrion. PfCIII is known to be inhibited by atovaquone [8,9,10]; DHODH is targeted by DSM-265 [26]; and, while the direct target of proguanil is not fully resolved, it is believed to disrupt the mitochondrial membrane potential, too [6,27]. Parasites with decreased levels of expression of the targets of those compounds are expected to be hypersensitive to their inhibition. This was demonstrated in a study of the *Plasmodium falciparum* mitochondrial ribosome (mitoribosome). Disruption of the mitoribosome leads to a decrease in functional PfCIII because one of the PfCIII components, cytb, is encoded in the mitochondrial genome, and indeed, such disruption led to hypersensitivity to atovaquone, DSM-265, and proguanil [28]. As we suspect that the depletion of PfRieske results in a decrease in functional PfCIII, we hypothesized that it would lead to similar hypersensitivity. Seeing that the growth defect following PfRieske depletion is detected on day six (third IDC), we added inhibitors on day four (at the ring stage of the second IDC) and monitored the parasite growth over the next cycle. Half Maximal Inhibitory Concentration (IC50) was then calculated on day six (Figure 5A). In line with our hypothesis, PfRieske-iKD parasites show hypersensitivity towards atovaquone, DSM-265, and proguanil (Figure 5B, Table S2). The IC50 for chloroquine and artemisinin, which do not interfere with the mETC, remained unchanged compared to the wild type (Figure 5C, Table S2), indicating that the observed hypersensitivity is specific to the inhibition of this pathway.

Since electron transport is coupled to proton pumping, which in *Plasmodium* is expected to occur in complexes III and IV, we reasoned that PfRieske depletion would reduce the mitochondrial membrane potential. We used MitoTracker-Deep-Red as a proxy for membrane potential and investigated the effect of PfRieske depletion and mETC inhibitors on its signal distribution. PfRieske-iKD parasites grown in the presence of GlcN for five days still presented MitoTracker-Deep-Red mitochondrial-specific staining, as did parasites treated with atovaquone (1 nM) or proguanil (1 µM) alone (Figure 5D,F). However, parasites treated with both atovaquone and proguanil lost the membrane potential-dependent mitochondrial staining of MitoTracker-Deep-Red, and the signal was detected in the cytosol instead (Figure 5E,F). Likewise, treatment of PfRieske-HA-glmS with both GlcN and proguanil resulted in the loss of the mitochondrial staining (Figure 5E,F). These data indicate that the depletion of PfRieske disrupts the membrane potential in a manner that mimics the disruption caused by inhibition with 1 nM of atovaquone in this assay.

### 2.4. PfRieske Is Required for the Pyrimidine Synthesis Pathway

**Figure 6:**
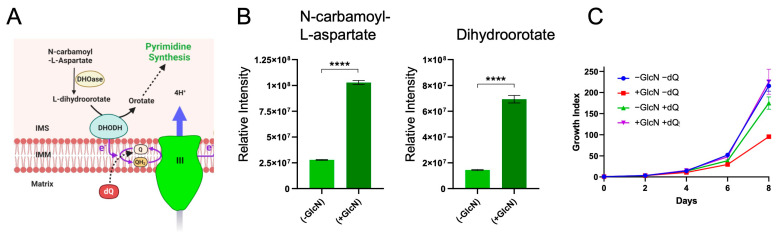
Accumulation of pyrimidine synthesis precursors in PfRieske-iKD, while treatment with decyl-ubiquinone rescues its growth defect: (**A**) schematic image of the part of the pyrimidine synthesis pathways involving DHODH, ubiquinone, and PfCIII. The mitochondrial intermembrane space (IMS), inner membrane (IMM), ubiquinol (QH2), ubiquinone (Q), and decyl-ubiquinone (dQ) are shown. (**B**) Bar graph showing the levels of the analyzed pyrimidine synthesis pathway intermediates, N-cabamoyl-L-aspartate (left) and dihydroorotate (right), in the PfRieske-iKD line grown in the presence or absence of GlcN (Rieske/3D7 (±GlcN). Unpaired *t*-test was used with **** being <0.0001 and error bars are SD. (**C**) Growth curve of PfRieske-iKD in the presence and absence of GlcN (±GlcN) and of 30 µM dQ (±dQ). Errors bars are SEM.

The profile of hypersensitivity to inhibitors and the observed disruption of the mitochondrial membrane potential upon PfRieske depletion is in line with a decrease in functional PfCIII in this mutant. Previous studies demonstrated that the main function of PfCIII in asexual stage parasites is in ubiquinone recycling for the DHODH-ubiquinone oxidoreduction cycle, which is an essential step in the pyrimidine synthesis pathway [6]. We thus hypothesized that the observed growth defect is due to a defect in the pyrimidine synthesis pathway. Since we observed the growth arrest in the third cycle (Figure 4B), we isolated whole-cell metabolites from the trophozoite stage of this cycle (on day five) and performed targeted metabolomic analysis for intermediates of the pyrimidine synthesis pathway that are found upstream of DHODH: N-carbamoyl-L-aspartate and dihydroorotate (Figure 6A). We found that the level of both metabolites was significantly enhanced (~4-fold increase) in the PfRieske-iKD compared to no GlcN treatment (Figure 6B), suggesting that they accumulate in this mutant.

Previous studies have demonstrated that the need for PfCIII to oxidize ubiquinol to ubiquinone for the cycle of DHODH-ubiquinone oxidoreduction can be bypassed through the addition of the ubiquinone analog decyl-ubiquinone (dQ) to the parasite growth media [16,29,30]. It would, therefore, be expected that the observed growth defect upon PfRieske depletion should be rescued with dQ. Accordingly, parasites grown in the presence of both GlcN and dQ showed restored growth comparable to parasites grown in the absence of GlcN (Figure 6C). Taken together, these data provide additional support that PfRieske is required for PfCIII function and suggest that the observed growth defect of asexual stages in our mutant is likely due to a defect in pyrimidine synthesis. 

### 2.5. Loss of PfRieske Causes Decrease in Cristae Density and Affects Gametocytes Maturation

**Figure 7:**
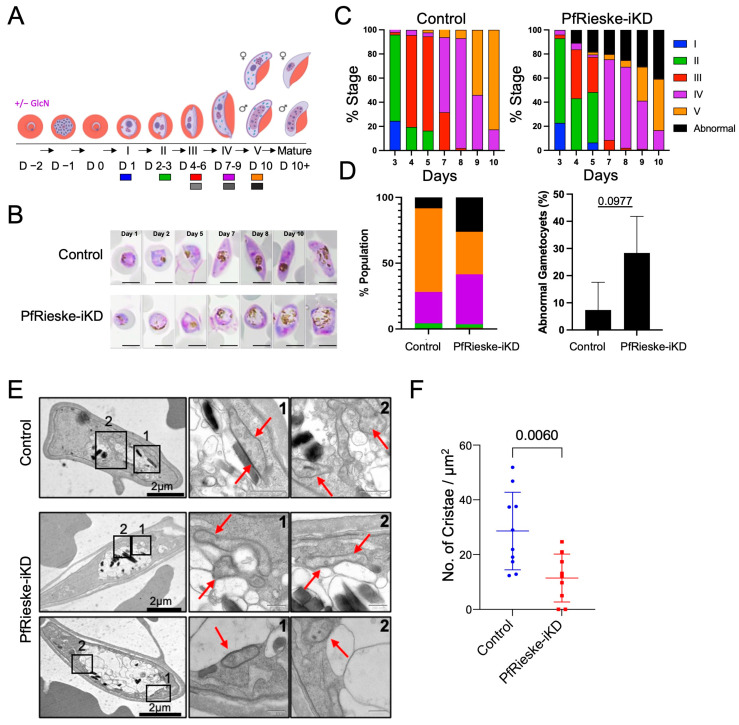
Knockdown of PfRieske arrests gametocyte development: (**A**) Schematics of the experimental setup of gametocyte induction in control and PfRieske-iKD along with a scheme of the different stages. (**B**) Progression of the control and PfRieske-iKD gametocytes monitored by Giemsa stained smears. Scale bar: 5 µm (**C**) One of three experiments following quantification of morphologies based on Giemsa-stained smears of control and Reiske-iKD gametocytes. (**D**) Quantification and analysis of progression numbers on day 10 of the three experiments. An unpaired *t*-test was used. (**E**) Transmission electron micrographs of the control and PfRieske-iKD gametocytes. Enlarged sections show mitochondria (red arrows). Scale bar: 200 nm. (**F**) Quantification of cristae density in the control and PfRieske-iKD gametocytes.

The transition from asexual stages to gametocytes is accompanied by a shift in carbon metabolism whereby gametocytes rely more on the TCA cycle, mETC, and ATP synthase for energy and metabolites [31,32]. In line with this shift, mETC components show an up to 40-fold increase in expression in the sexual stages [15]. We, therefore, expect that PfCIII will play a critical role in more pathways in the gametocyte stages compared to the asexual stages. This is supported by the finding that development in mosquitos and transmission depends on a fully functional PfCIII [21,33,34]. We were, therefore, interested in examining the impact of PfRieske depletion on gametocyte development. We induced gametocyte commitment using the spent-medium protocol [35] (Figure 7A,B). In three independent experiments, morphological analysis revealed that gametocyte development was affected by PfRieske depletion. In all three experiments, we found a consistent trend of increase in the percentage of “abnormal” gametocytes in PfRieske-iKD compared to the control, alongside a decrease in the percentage of parasites that reach stages IV/V (Figure 7B–D). Abnormal gametocytes were characterized by round overall morphology and what seemed to be a swollen food vacuole (Figure 7B,E). While consistent, this observed trend was not significant (Figure 7D), potentially due to variability in gametocytogenesis efficiency between our experiments.

Mitochondrial cristae shape is tightly linked to oxidative phosphorylation (OXPHOS) activity. Accordingly, the metabolic shift in gametocytes coincides with the de novo appearance of cristae [7,36,37]. We thus hypothesized that the defect in gametocyte development upon PfRieske depletion would be linked to cristae abnormalities. In support of this hypothesis, examination of the PfRieske-iKD parasites using transmission electron microscopy (TEM) revealed abnormal mitochondrial morphology upon PfRieske depletion (Figure 7E). Quantification of cristae numbers per mitochondrial area further showed that these parasites have a decreased cristae density in their mitochondria compared with their parental line (Figure 7F). To our knowledge, this is the first time that a cristae defect is observed upon disruption of a *Plasmodium* gene. These data show that PfRieske is required for maintaining the cristae density in *P. falciparum* gametocytes which may ultimately help the parasite in fulfilling the metabolic needs required for gametocyte maturation.

## 3. Discussion

The mETC is critical for the survival of many eukaryotic cells, including both the apicomplexans and their hosts. Nevertheless, the parasite pathway shows different sensitivity to inhibitors compared to the human one. For this reason, the *Plasmodium* mETC has been the focus of numerous drug discovery studies for antimalarial drug development, including a recent screen of the Medicines for Malaria Venture (MMV) “pathogen box” [38]. Most of our knowledge about *Plasmodium* mETC complexes, including PfCIII, is, therefore, based on studies using inhibitors or analyzing drug resistance, while their role within parasite biology is less well understood. Here, we used genetic disruption to provide an improved understanding of the role of PfCIII in parasite biology in asexual and sexual stages.

Recent studies in apicomplexans proposed a divergent set of mETC complex subunits compared to the mETC of the host and highlighted new parasite subunits in each of the complexes [15,17,39,40]. PfCIII was proposed to consist of 12 subunits with a predicted higher molecular size compared to human complex III [15]. Using C-terminal tagging, we provided support for this prediction by recovering some of the new subunits in immunoprecipitation experiments and via BN-PAGE and Western blot analysis, which confirmed that both the conserved PfRieske and the new subunit PfC3AP2 are part of a complex which migrates at size of ~730 kDa. It will be of interest to understand how the new subunits contribute to PfCIII function and what is their mechanism of action in this important complex. One possibility is that they would structurally “fill-in” for the missing homologs of the non-catalytic subunits found in other systems, as we recently proposed for the non-catalytic subunits of mETC complex II in the related *T. gondii* parasite [40]. Another option is that they may mediate interaction with other mETC complexes to form supercomplexes, as suggested for non-catalytic and species-specific subunits in other systems [41]. Importantly, regardless of their function, the presence of different proteins in PfCIII compared to its human counterpart may well induce small changes in the location and orientation of amino acids in and around the substrate binding pockets that could affect the interaction of PfCIII with inhibitors, which may provide opportunities for new drug development. Structural analysis of PfCIII will likely help shed light on these open questions.

Considering the essential role of PfCIII demonstrated in previous inhibitor studies, it is expected that its subunits will be important for parasite survival. In agreement with this expectation, a previous study shows that a conditional depletion of mACP, involved in iron-sulfur cluster biogenesis, leads to secondary loss of PfRieske, ultimately leading to asexual stage parasite death [30]. Likewise, the depletion of cytC1 protein demonstrated its essentiality for mETC functions and the growth of asexual stages [16]. Our conditional knockdown adds support to these observations by showing directly that PfRieske is essential for asexual growth. However, to our surprise, PfC3AP2 protein downregulation did not have a significant impact on growth. This was unexpected given that in the genome-wide piggy-back transposons-based study, PfC3AP2 is predicted to be essential for growth [42], and our previous study of the PfC3AP2 homolog from *T. gondii* also showed that it is essential for parasite survival [17]. We cannot fully conclude whether the regulation accomplished with the glmS system is insufficiently tight or whether PfC3AP2 is truly dispensable for asexual blood-stage parasites at this point.

Previous studies demonstrated that disruption of a component of PfCIII [16] and of the *P. falciparum* mitoribosome [43] (required to translate the essential PfCIII subunit cytb), leads to parasites hypersensitive for mETC inhibitors, likely since there is less PfCIII activity remaining to inhibit in those mutants. Studies further suggested that a potential alternate pathway to polarize the inner mitochondria membrane independent of PfCIII and complex IV exists in *Plasmodium* [6,44]. This pathway can be blocked by proguanil only in the presence of a PfCIII inhibitor. We observed that loss of PfRieske alone did not result in membrane depolarization, which instead required a combination of PfRieske depletion and treatment with proguanil. Collectively, our observations thus agree with previous findings, thus providing support that PfRieske depletion results in PfCIII disruption in our case, too.

The mETC plays two major cellular functions: One is electron transfer, whereby the transported electrons reduce a final electron acceptor, often oxygen. This electron transfer function enables different metabolic pathways through the oxidation of key enzymes, such as DOHOD of the pyrimidine synthesis pathway; the other is oxidative phosphorylation, whereby the electrons transported from TCA cycle metabolites are coupled to the pumping of protons by mETC complexes, generating an electrochemical gradient that facilitates ATP synthesis by ATP synthase. The *P. falciparum* life cycle is complex, consisting of multiple developmental stages and requiring survival in two hosts. The different stages and the movement between host environments manifest in major metabolic shifts, and the reliance on different roles of the mETC is expected to vary accordingly. For example, it was established that in the asexual blood stages, the main function of *P. falciparum* mETC is enabling the oxidation of DHODH for de novo pyrimidine synthesis [6]. Our metabolomic data confirm that PfRieske depletion results in a defect in the pyrimidine synthesis pathway and the accumulation of two key metabolites downstream of DHODH. Likewise, the ability of dQ to rescue the growth of PfRieske depleted parasites, which is in line with previous observations [16,29], provides further support for the sole role of ubiquinone recycling that PfCIII plays in the asexual blood-stages grown in cultures.

On the other hand, different findings point to the importance of active oxidative phosphorylation in the transmissible gametocyte stages, including the increased expression of genes encoding mETC components in gametocytes [15] and the growth defect of different life stages upon disruption of other mETC enzymes such as complex II, ATP synthase and NDH2 in *P. berghei* [7,45,46]. Likewise, mitochondrial cristae, which are a hallmark of active oxidative phosphorylation, are almost only present in the sexual stage parasites among the blood stage parasites. In line with these observations, we found a defect in gametocyte development upon PfRieske depletion, and this outcome coincides with reduced cristae density in those PfRieske-depleted gametocytes. In another protozoan, *Tetrahymena thermophila*, complex III is part of a supercomplex that plays a role in curving the cristae [47]. It is possible that one of the PfCIII-containing supercomplexes that were detected in *Plasmodium* and that were found to be more abundant in sexual compared with asexual stages [15] plays a similar role in these parasites. In this scenario, PfCIII depletion would lead to a decrease in the number of any supercomplexes involving it and, thus, to the observed cristae formation defect. However, the mechanism controlling cristae biogenesis in *P. falciparum* requires further studies at this point.

Interestingly, on top of the mitochondrial ultrastructure defect, PfRieske-depleted gametocytes also show an enlarged food vacuole. Seeing that the food vacuole plays a central role in parasite metabolism via hemoglobin degradation and recycling of amino acids, we speculate that the functions of these two organelles may be coordinated and that the two phenotypes are potentially linked. The apicomplexan mitochondrion is seen to be involved in active membrane contact sites with several organelles (ER, pellicles, nucleus [48,49,50,51,52]). It would be of interest to explore if there are contacts with the food vacuole, too.

## 4. Materials and Methods

### 4.1. Plasmid Construction, Parasite Culture, Transfection, and Transgenic Line Confirmation

To generate the HA-glmS [18] construct, the C-terminal regions of PfRieske and PfC3AP2 were PCR amplified using (Table 2) sets (2448, 2449 for C3AP2 and 2451, 2535 for Rieske) and cloned into HA-glmS vector in *BglII* and *PstI* restriction sites. *Plasmodium falciparum* 3D7 strain was cultured under standard culture conditions in RPMI media supplemented with 0.5% (*w*/*v*) Albumax (Invitrogen, Paisley, UK). To generate the HA-glmS transgenic parasite lines, 3D7 parasites were synchronized repeatedly by sorbitol treatment, and 100 μg of plasmid DNA for the respective gene was transfected in *P. falciparum* by electroporation (0.310 kV and 950 μF) [53]. Transfected parasites were selected with WR 99210 (Jacobus Pharmaceuticals, Plainsboro Township, NJ, USA). The clonal selection was performed by serial dilution in a 96-well plate, and the integration was confirmed by PCR using (Table 2) set (2718, 2797 for C3AP2 and 2719, 2797 for Rieske) and by Western blot analysis using an anti-HA antibody (Roche, London, UK).

### 4.2. Inducible Knockdown and Parasite Growth Assay

Both the transgenic parasite lines were tightly synchronized with repeated 5% sorbitol treatment. To assess the knockdown at the protein level, 2.5 mM glucosamine (GlcN) (Sigma, Glasgow, UK) was added at the ring stage, and schizont stage parasites were harvested using saponin. The protein samples for Western blotting were prepared from these parasites. For the growth assay, parasites were cultured with/without GlcN conditions for several cycles, and the parasitemia was assessed at different intervals by malaria SYBR Green I-based fluorescence assay (MSF) using MACSQuant. Briefly, cells were incubated with SYBR green for 20 min at 37 °C in the dark, followed by the 3 washings with 1 X PBS, and these samples were analyzed via the MACSQuant system. For each set, 100,000 cells were counted to monitor the parasitemia. Uninfected RBCs were used as a negative control. To assess the stage-specific knockdown effect, Giemsa (Sigma)-stained smears were prepared at different intervals from the control and GlcN-treated parasites and analyzed by light microscope. Thousand infected RBCs (iRBCs) were counted to check the stage-specific defect of the PfRieske-iKD.

### 4.3. Immunoprecipitation and Mass Spectrometry

PfRieske-HAglmS or parental 3D7 parasite pellets were resuspended in lysis buffer (150 mM NaCl, 2 mM EDTA, 50 mM Tris-HCl pH 7.4, 1% (*w*/*v*) βDDM, protease inhibitor) and incubated at 4 °C for 2 h. Samples were then centrifuged at 16,000× *g* for 30 min at 4 °C to separate soluble and insoluble material. The supernatant, containing solubilized intact protein complexes, was taken and incubated with anti-HA agarose conjugated agarose beads (Pierce) overnight at 4 °C. Beads were washed twice with a buffer containing 0.05% (*w*/*v*) βDDM. Bound proteins were then eluted with 50 mM NaOH and sent for analysis by mass spectrometry.

An additional experiment was performed on a mitochondrially enriched sample of PfRieske-HAglmS parasites. Briefly, eight large dishes of 10% late trophozoite-schizont stage parasites were harvested using the 0.15% saponin treatment. Parasite pellets were washed with 1× PBS to remove hemoglobin contamination before cell lysis using nitrogen cavitation at 1500 p.s.i. for 20 min. Lysate was passed through the Miltenyi Biotech MACS column to remove the hemozoin, and mitochondrial material was enriched through centrifugation at 16,000× *g* for 30 min at 4 °C. PfCIII was immunoprecipitated as above, with the elution performed instead by incubation overnight with HA peptide (1 mg/mL), and samples sent for analysis by mass spectrometry.

For mass spectrometry analysis, trypsin-digested peptide samples were prepared, and solubilized in 20 µL 5% acetonitrile with 0.5% formic acid using the auto-sampler of a nanoflow uHPLC system (Thermo Scientific, RSLCnano, Perth, UK). Online detection of peptide ions was by electrospray ionization (ESI) mass spectrometry MS/MS with an Orbitrap Elite MS (Thermo Scientific). The ionization of LC eluent was performed by interfacing the Sharp Singularity emitters (Fossil Ion Tech, Madrid, Spain) with an electrospray voltage of 2.5 kV.

An injection volume of 5 µL of the reconstituted protein digest was desalted and concentrated for 1.1 min on a trap column (0.3 × 5 mm) using a flow rate of 25 µL/min with 1% acetonitrile with 0.1% formic acid. Peptide separation was performed on a Pepmap C18 reversed-phase column (50 cm × 75 µm, particle size 3 µm, pore size 100 Å, Thermo Scientific) using a solvent gradient at a fixed solvent flow rate of 0.3 µL/min for the analytical column. The solvent composition was (A) 0.1% formic acid in water and (B) 0.08% formic acid in 80% acetonitrile 20% water. The solvent gradient was 4% B for 1.5 min, 4 to 60% for 100.5 min, 60 to 99% for 14 min, and held at 99% for 5 min. A further 9 min at initial conditions for column re-equilibration was used before the next injection. Protein identifications were assigned using the Mascot search engine (v2.6.2, Matrix Science, https://www.matrixscience.com/, accessed on 6 May 2024) to interrogate protein sequences in the *Plasmodium falciparum* 3D7 database. A mass tolerance of 10 ppm was allowed for the precursor and 0.3 Da for MS/MS matching. Proteins detected in the negative control (3D7) were subtracted as background contaminants. For a full list of all proteins detected, see Table S1.

### 4.4. Denaturing Polyacrylamide Gel Electrophoresis (SDS-PAGE), Blue Native (BN-PAGE), and Western Blotting

For SDS-PAGE, Parasites were isolated by lysing the infected RBCs with 0.15% saponin, and the parasites were resuspended in Laemmli buffer and lysed by heating at 95 for 10 min. Proteins were further separated on the 12% SDS-PAGE and transferred to the PVDF or nitrocellulose membrane (Millipore, Glasgow. UK) using a semi-dry trans-blot (Bio-Rad, London, UK). For the BN-PAGE, samples were prepared by resuspending the protein samples in a solubilization buffer, incubated on ice for 30 min, and centrifuged at 16,000× *g* at 4C for 30 min. The supernatant was combined with a sample buffer containing Coomassie G250 (NativePAGE) at the final concentration of 0.25% DDM and 0.0625% Coomassie G250. The samples were separated on the 4–16% Bis-Tris native gel and transferred to the PVDF membrane.

For the Western blotting, membranes were incubated with a blocking solution (5% skimmed milk in 1× PBS) followed by overnight incubation with respective primary antibodies (anti-aldolase rabbit 1:2000 (Abcam, Cambridge, UK); anti-HA rat 1:1000 (Roche, London, UK); anti-BiP rabbit 1:5000) in a cold room and 1 hr incubation with respective secondary antibodies (1:10,000) at room temperature and visualized by using ECL kit (Thermo Scientific).

### 4.5. Immuno-Fluorescence Assay (IFA) and Microscopy

Infected RBCs (5–10% parasitemia) were fixed in 4% paraformaldehyde and 0.05% glutaraldehyde for 50 min at room temperature on a rotator. Cells were permeabilized by 10 min incubation with 0.1% Triton X-100 on ice and incubated with blocking buffer (10% FBS in 1× PBS) for 90 min and then probed with the primary antibody anti-HA rat 1:100 (Roche) and anti-Pfs25 rabbit 1:1000 (BEI Resources, https://www.beiresources.org/, accessed on 6 May 2024) for 2 h on the rotor and after three washing with 1× PBS cells were probed with anti-rat Alexa Flour-488 (Promega, Chilworth, UK) 1:500 for 1 h. Parasite nuclei were stained with Hoechst with a final concentration of 5 μg/mL. Images were captured by a Nikon A1 confocal laser scanning microscope and analyzed by Nikon-NIS element software (version 4.1).

To label mitochondria, infected RBCs were incubated with 100 nM MitoTracker-Deep-Red (Thermo Scientific) for 15 min at 37 °C shaker incubator and washed with 1× PBS thrice. Cells were further fixed and processed for IFA, as described earlier.

### 4.6. Metabolomic Profiling

Rieske-HAglmS parasites were synchronized, and the knockdown assay was set up as described earlier. Parasites were isolated from the control and PfRieske-iKD parasites using saponin, and 3 × 10^8^ number of parasites were used for metabolites isolation. Isolated parasites were quenched by rapidly cooling the cells to 4 °C by dry ice/ethanol. Metabolites were isolated using the chloroform/methanol/water (1:3:1) method. Briefly, cells were resuspended in 600 µL of chloroform/methanol/water (1:3:1) and kept on vortex for 1 hr at 4 °C. After centrifugation for 3 min at 13,000× *g* at 4 °C, the supernatant was transferred into new Eppendorf tube and stored in −80 °C until analysis by LC-MS.

Hydrophilic interaction liquid chromatography (HILIC) was carried out on a Dionex UltiMate 3000 RSLC system (Thermo Fisher Scientific, Hemel Hempstead, UK) using a ZIC-pHILIC column (150 mm × 4.6 mm, 5 μm column, Merck Sequant, Glasgow, UK). The injection volume was 10 μL, and samples were maintained at 5 °C prior to injection. For the MS analysis, a Thermo Orbitrap QExactive (Thermo Fisher Scientific) was used.

Metabolomics samples from the control samples (3D7 ± GlcN, 3D7 treated with ATQ) were also prepared and analyzed in the same way.

### 4.7. Drug Sensitivity Assay

The drug assay was performed in a 96-well plate. Rieske-HA parasites were grown in media with/without GlcN for two cycles, and second-cycle ring parasites were exposed to drugs diluted in serial dilution. In the third cycle, parasites were lysed in SYBR green lysis buffer, and fluorescence intensity was assayed using a spectrophotometer. IC_50_ graphs for drugs were prepared using Graph Prism 10 software. All the compounds used in this assay were purchased: atovaquone, DSM-265, proguanil, Chloroquine, and Artemisinin (Med Chem Express, Monmouth Junction, NJ, USA, Arcos Organics, Perth, UK and Sigma Aldrich, Glasgow, UK, respectively).

### 4.8. Transmission Electron Microscopy

For electron microscopy analysis, control and PfRieske-iKD gametocytes were fixed with prewarmed 2% glutaraldehyde in a 0.1 M cacodylate (pH 7.4) buffer. Following serial washes in 0.1 M phosphate buffer, samples were post-fixed in 1% OsO4 and 1.25% potassium ferrocyanide (*v*:*v*) in the same buffer for 1 h in the dark, washed with distilled water and contrasted EM bloc with 0.5% aqueous uranyl acetate for 1 h at room temperature in the dark. The samples were then dehydrated in acetone ascending series (30%, 50%, 70%, 90%, 100%) and embedded in epoxy resin. Ultra-thin sections (50 nm) were sectioned in a Leica Ultramicrotome and collected in 100 mesh grids covered with formvar. The collected sections were contrasted with 2% aqueous uranyl acetate and later observed in a Jeol 1200 transmission electron microscope (Jeol, Tokyo, Japan) operating at 80 kV.

### 4.9. Gametocyte Commitment and Culturing

Gametocyte commitment assay was induced by stress using spent media. Briefly, Rieske-HA-glmS parasites were synchronized and divided in GlcN (+) or GlcN (−) conditions one cycle before the commitment. Gametogenesis commitment was induced using nutritional stress by growing parasites with spent medium (day two) and then growing parasites in media having heparin (1:500) till day +three to block the invasion of the asexual stage parasites. After that parasites were cultured with normal media conditions. Giemsa-stained smears were prepared at different time intervals for the morphological analysis.

## Figures and Tables

**Table 1:** The interacting partners of the PfRieske identified via immunoprecipitation and mass spectrometry analysis. Full MS data are in Table S1.

Expected	Detected (Epx1)	Detected (Exp 2)	Accession
Cytb	Yes	Yes	PF3D7_MITO2300
Cytc1	Yes	Yes	PF3D7_1462700
Rieske	Yes	Yes	PF3D7_1439400
QCR6	No	No	PF3D7_1426900
QCR7	Yes	Yes	PF3D7_1012300
QCR8	No	No	PF3D7_0306000
QCR9	No	Yes	PF3D7_0622600
MPPalpha	Yes	Yes	PF3D7_0523100
MPPbeta	Yes	Yes	PF3D7_0933600
C3AP1	No	Yes	PF3D7_0722700
C3AP2	Yes	Yes	PF3D7_1326000
C3AP3	Yes	Yes	PF3D7_0817800

**Table 2:** Primers unsed in this work.

Name	Sequence
2448 PfC3AP2-FWD	GCAGATCTCCTTCAGATAGTAGGAAATTTG
2449 PfC3AP2-REV	GCCTGCAGC TGCGTGTTCAACTGA
2535 PfRieske-FWD-B	GCGGATCCCCTGTATTTCCAAGAAAACC
2451 PfRieske-REV	GCCTGCAGCTCCAATTTTTATCGTATTTTC
2718 PfC3AP2-Int1	CAAAAGAATTCAGGGTAAATTTATACCTTG
2719 PfRieske-Int1	GTATTCCTCCTGCATCTGAAGATC
2797 HAglmS-REV-1	TGATCTGCACACTCAGCCGGGA

## Data Availability

Data is contained within the article and Supplementary Materials.

## Supplementary Materials

The following supporting information can be downloaded at https://www.mdpi.com/article/10.3390/ijms25179239/s1.
