# Supplementary material for: Plasmodium falciparum Mitochondrial Complex III, the Target of Atovaquone, Is Essential for Progression to the Transmissible Sexual Stages
Source: Int J Mol Sci. 2024 Aug 26;25(17):9239. doi: 10.3390/ijms25179239 (PMC11394760; doi:10.3390/ijms25179239)
Supplement: Supplementary file 1 [file ijms-25-09239-s001.zip › Figure S1.pdf]

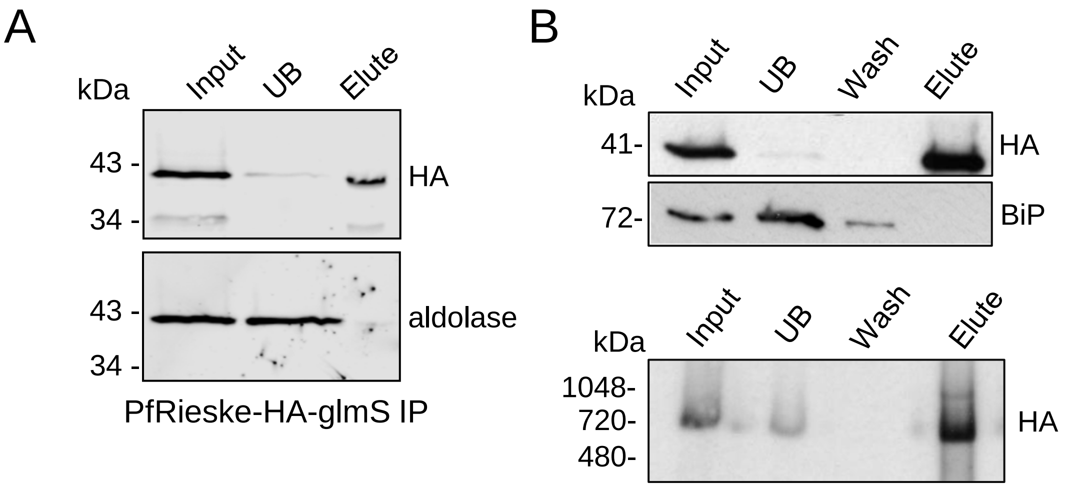

Figure S1. (A) SDS-PAGE and immunoblot analysis of immunoprecipitation fractions from whole-cell PfRieske-HA-glmS sample. Aldolase was used as a fraction specificity control. Input- whole cell sample; UB- unbound material. **(B)** SDS (top panels) and native (bottom panel) PAGE and immunoblot analysis of immunoprecipitation fractions from mitochondrially-enriched PfRieske-HA-glmS sample, showing the HA enrichments and the high migrating complex found in the elute. BiP was used as fraction specificity control. Input – enriched mitochondria.
